# Position-dependent trunk asymmetry assessed with scoliometer

**DOI:** 10.1186/1748-7161-7-S1-O60

**Published:** 2012-01-27

**Authors:** J Chowańska, T Kotwicki, K Rosadziński

**Affiliations:** 1 Rehasport Clinic, Poznań, Poland; Spine Disorders Unit, Department of Pediatric Orthopedics and Traumatology, University of Medical Sciences, Poznan, Poland; 2 Spine Disorders Unit, Department of Pediatric Orthopedics and Traumatology, University of Medical Sciences, Poznan, Poland

## Purpose of the study

It was to assess the influence of child positioning during examination with Bunnell scoliometer on value of angle of trunk inclination.

## Background

During school screening for scoliosis the Angle of Trunk Inclination/Rotation (ATI/ATR) measurement is performed with the use of scoliometer. Early adolescent girls are the target group for scoliosis screening. Further evaluation is recommended when ATI is equal or above 7°. Standing forward bending position is a standard one, however sitting position is also advocated [1-7].

## Materials and methods

The study comprised 996 girls, aged 9 to 13, mean 11.0 ± 1.0 years of age. ATI measurements were performed at three levels of the spine: proximal thoracic, main thoracic and thoracolumbar/lumbar. Maximal ATI values for standing and sitting forward bending positions were noted. Based on the ‘7°’ criterion, the number of children who need follow up was revealed, according to position: (a) standing, (b) sitting, (c) any of standing or sitting, (d) either standing or sitting.

## Results

On each level of the spine the ATI value was lower for the sitting forward-bending position than for the standing one. The prevalence of Bunnell ≥ 7° was as follows: (a) 3.9%, (b) 3.2%, (c) 4.5% and (d) 2.4%.

## Conclusions

The value of ATI depends on body position during scoliometer measurement. Sitting position can be considered for the purpose of school screening for scoliosis, alone or as complement of the standing one.
